# Superior cancer preventive efficacy of low versus high dose of mTOR inhibitor in a mouse model of prostate cancer

**DOI:** 10.18632/oncotarget.27550

**Published:** 2020-04-14

**Authors:** Marina P. Antoch, Michelle Wrobel, Bryan Gillard, Karen K. Kuropatwinski, Ilia Toshkov, Anatoli S. Gleiberman, Ellen Karasik, Michael T. Moser, Barbara A. Foster, Ekaterina L. Andrianova, Olga V. Chernova, Andrei V. Gudkov

**Affiliations:** ^1^Department of Pharmacology and Therapeutics, Roswell Park Comprehensive Cancer Center, Buffalo, NY, USA; ^2^Everon Biosciences, Inc., Buffalo, NY, USA; ^3^Cell Stress Biology, Roswell Park Comprehensive Cancer Center, Buffalo, NY, USA

**Keywords:** prostate cancer, rapamycin, prevention, mTOR, PTEN

## Abstract

The mechanistic target of rapamycin (mTOR) is a PI3K-related kinase that regulates cell growth, proliferation and survival in response to the availability of energy sources and growth factors. Cancer development and progression is often associated with constitutive activation of the mTOR pathway, thus justifying mTOR inhibition as a promising approach to cancer treatment and prevention. However, development of previous rapamycin analogues has been complicated by their induction of adverse side effects and variable efficacy. Since mTOR pathway regulation involves multiple feedback mechanisms that may be differentially activated depending on the degree of mTOR inhibition, we investigated whether rapamycin dosing could be adjusted to achieve chemopreventive efficacy without side effects. Thus, we tested the efficacy of two doses of a novel, highly bioavailable nanoformulation of rapamycin, Rapatar, in a mouse prostate cancer model (male mice with prostate epithelium-specific *Pten*-knockout). We found that the highest efficacy was achieved by the lowest dose of Rapatar used in the study. While both doses tested were equally effective in suppressing proliferation of prostate epithelial cells, higher dose resulted in activation of feedback circuits that reduced the drug’s tumor preventive efficacy. These results demonstrate that low doses of highly bioavailable mTOR inhibitor, Rapatar, may provide safe and effective cancer prevention.

## INTRODUCTION

Prostate cancer (PCa) is the second-leading cause of death from cancer in men. 191,930 new cases of and 33,330 deaths from PCa are estimated for the United States alone during 2020 [1]. The initial anticancer effect of the standard treatment, androgen deprivation, is commonly followed by development of recurrent castration-resistant disease that is frequently lethal [2]. Therefore, in addition to developing therapeutic approaches, it is vitally important to advance strategies for prevention of PCa.

Although epidemiological and clinical data provide evidence for a potential role for dietary factors in PCa development, results remain inconclusive (reviewed in [3]). A randomized clinical trial testing the effect of selenium, vitamin E or their combination on the risk of development of PCa in healthy males was terminated prematurely due to initial results showing no effect of selenium alone or in combination with vitamin E [4]. Moreover, dietary supplementation with vitamin E significantly increased the risk of PCa among healthy men [5]. Two randomized clinical trials that tested inhibitors of 5α-reductase (finasteride and dutasteride) for PCa prevention showed a reduction in tumor incidence, but no effect on mortality [6, 7]. In addition, men in the finasteride-treated group had more aggressive tumors and increased sexual dysfunction [7]. Thus, new approaches to PCa prevention are needed.

The mechanistic target of rapamycin, mTOR, is a serine/threonine kinase that regulates cell metabolism, growth and proliferation in coordination with various environmental factors, such as availability of nutrients and growth factors {Condon, 2019 #431}. MTOR functions in two distinct complexes, mTORC1 and mTORC2, which have different downstream targets. MTORC1 phosphorylates targets involved in protein biosynthesis including ribosomal protein S6 kinase (S6K) and eukaryotic initiation factor 4E (eIF-4E) -binding protein 1 (4E-BP1) [8]. On the other hand, mTORC2 promotes cell cycle progression and survival by phosphorylating Akt at Ser473 [9]. The phosphatidylinositol 3-kinase (PI3K)/Akt/ mTOR pathway plays a critical role in the development of PCa and progression to castration-resistant PCa: components of the PI3K/Akt/mTOR pathway are altered in 42% of primary and 100% of metastatic PCa cases [10]. Moreover, deregulation of this pathway is often linked to resistance to cancer therapy [11].

Phosphatase and tensin homolog (PTEN) is a tumor suppressor that acts as a negative regulator of PI3K/Akt/mTOR-mediated signaling [12]. PTEN was shown to be the most commonly lost tumor suppressor in primary PCa [6] and its loss correlates with both pathological stage of the disease [13] and rate of metastasis [14]. At the molecular level, loss of PTEN results in increased levels of mTOR and its substrates 4E-BP1 and S6 in prostate tissue [15]. Taken together, these findings strongly suggest that inhibitors of the PI3K/Akt/mTOR pathway may be effective preventive and therapeutic agents in PCa.

Among know inhibitors of PI3K/Akt/mTOR signaling, the mTOR inhibitor rapamycin is of special interest. It is a macrolide antibiotic that was first isolated from *Streptomyces hydroscopicus* and initially used clinically as an antifungal agent [16]. Under the name of Rapamune (sirolimus), it is currently used as an immunosuppressant to prevent organ rejection after transplantation [17, 18]. Recent data demonstrated that rapamycin extends life span in various model organisms, including mammals [19–21]. Life-long administration of rapamycin inhibits age-related weight gain, decreases the rate of aging, and increases the lifespan of inbred and genetically heterogeneous mice [21]. Importantly, administration of rapamycin significantly delays the onset of spontaneous carcinogenesis in both normal (129/Sv [22]) and cancer-prone HER-2/neu transgenic [23] and *p53^+/–^* knockout [24] mice. Treatment with rapalogs also reversed Akt-induced prostatic intraepithelial neoplasia (PIN) phenotype in the model of transgenic mice expressing human AKT1 in the ventral prostate (AKT1-Tg) [25].

Although the results of multiple reports identified rapamycin as perspective chemopreventive drug for clinical use, they also revealed significant shortcomings. First, rapamycin exhibits poor water solubility and instability in aqueous solutions, therefore its clinical use through oral administration requires modifications in drug design and/or formulation to increase bioavailability and efficacy. For example, one rapamycin derivative, everolimus, was designed to bear a stable 2-hydroxyethyl chain substitution to increase its polarity, improve pharmacokinetic characteristics and increase bioavaibility [26]. However, even this optimized version of rapamycin was found to cause some adverse effects including hypertriglyceridemia, hypercholesterolemia, opportunistic infections, thrombocytopenia and leukocytopenia [26]. Secondly, poor water solubility and bioavailability require to use very high doses of the drug (in various mouse models between 10 and 40 mg/kg) to achieve either therapeutic or preventive effect or use prolonged treatment schedules, which resulted in development of serious side effects. Thus, prolonged treatment with rapamycin was reported to increase mortality in a mouse model of type 2 diabetes [27] and was also associated with increased incidence of diabetes when used as an immunosuppressor in renal transplantation [28] and sometimes with dermatological complications [29]. Therefore, despite notable beneficial effects, the high incidence of adverse side effects limits the use of rapamycin-based mTOR inhibition as either a chemopreventive or therapeutic approach [30]. These limitations are most likely due to the complexity of the entire mTOR signaling pathway and the existence of numerous feedback loops that may be activated in response to mTOR inhibition [31].

In our previous work we showed that a novel water-soluble and orally bioavailable nanoformulation of rapamycin named Rapatar effectively delayed carcinogenesis and increased lifespan in highly tumor-prone *p53^–/–^* mice [32]. Rapatar was also found to decrease chemically induced benign prostate hyperplasia (BPH) in rats [33]. Given its previously demonstrated efficacy and safety, we sought to test whether Rapatar could be used at low doses (below those inducing adverse effects) as an effective chemopreventive agent against prostate cancer. Based on our previous data, we choose to use Rapatar at the dose of 25 mg/kg (corresponds to 0.5 mg/kg of rapamycin), which was shown to delay carcinogenesis in the tumor-prone *p53^–/–^* mice [32] and even a lower one (5 mg/kg; corresponds to 0.1 mg/kg of rapamycin). Using a model in which mice with prostate-specific deletion of *Pten* (*psPten–/–*) spontaneously develop PCa, we demonstrated that chronic oral administration of Rapatar at these two doses in fact, suppresses tumorigenesis. Histological analysis of prostate tissue showed that Rapatar treatment resulted in reduced proliferation of prostate epithelial cells at both doses tested. Further evaluation of the overall severity of prostate tumor development as a combination of multiple parameters (tumor burden, development of reactive stroma, and presence of immune cell infiltration) demonstrated that the better level of protection was achieved by the low dose of Rapatar (5 mg/kg). Higher dose of 25 mg/kg was accompanied by development of reactive tumor stroma, induction of autophagy, and activation of Akt (leading to activation of pro-survival pathways), which would be expected to counteract its suppressive effect on prostate cell proliferation. Overall, our data supports an unexpected and simple solution for improving PCa prevention: partial inhibition of mTOR by low doses of Rapatar.

## RESULTS

### Morphological evaluation and scoring of PCa development in prostate-specific Pten–/– mice

To identify the optimal time for starting Rapatar treatment, we evaluated the dynamics of spontaneous PCa development in *psPten–/–* mice by histopathological evaluation of prostate tissue samples from 71 mice of different ages (ranging from 6 to 30 weeks) collected at various stages of tumor development. Previous studies demonstrated that *psPten–/–* mice start developing multifocal hyperplasia from 4 weeks on. This is followed by PIN formation starting at the age of 6 weeks, which further on develops to adenocarcinoma [34]. The progression of morphological changes with time was graded using a semi-quantitative scoring system (scores of 0 to 5) to assess the degree of hyperplasia and dysplasia/neoplastic growth. Our grading system is a slight modification of the histologic grading system used by Gingrich et al. for the TRAMP model [35], in which we took into consideration the recommendations of human and veterinary pathologists working with models of PCa [35–37], the guidelines of the Gleason grading system [38], and our own experience with scoring the severity of pathohistological changes induced by radiation or infectious or toxic agents in various tissues and organs. The following criteria were used for scoring of prostate tissue samples:

Score of 0: normal morphology of acini with simple secretory epithelium with cuboidal or columnar cells, regular clear nuclei and pale eosinophilic cytoplasm surrounded by sparse fibromuscular stroma;

Score of 1: mildly abnormal morphology of the epithelial lining, showing some hyperplasia and single sites of proliferation of epithelial cells with normal morphology;

Score of 2: moderately abnormal morphology, including interepithelial hyperplasia of epithelial cells resulting in thicker walls of acini, minimal protrusions into the lumen, and rounded or stellate transverse sections; protrusions do not display infolding of the basal membrane or blood vessel branches nor accumulations of cells;

Score of 3: markedly abnormal morphology, including presence of papillary infoldings and prominent intraluminal ridges of hyperplastic epithelium, partial obstruction of the lumen of acini/tubules with asymmetry of the epithelial lining (flatter on one side than the other); hyperplasia is confined within the surrounding thickened stromal layer;

Score of 4: severely abnormal morphology, including presence of densely packed hyperchromatic cells of atypical appearance, numerous mitotic figures and apoptotic bodies resulting in complex and disorderly clustering of glands in a cribriform pattern with indistinguishable lumen; samples display cell crowding, stratification, nuclear enlargement and pleomorphism of nuclei and cells (indicators of malignant transformation), expansion of the fibromuscular stroma layer, and mononuclear cell infiltration in the interstitium;

Score of 5: drastically abnormal morphology characteristic of poorly differentiated invasive adenocarcinoma, including presence of neoplastic cells that are highly variable in shape and size, have clumped chromatin, and form nests and sheets with no glandular architecture, aggressive neoplastic growth breaking through the fibromuscular stroma spreading locally to adjacent lobes or to pelvic lymph nodes.

Examples of the various stages of PCa development in the *psPten–/–* mouse model and their associated scores using the system described above are presented in Supplementary Figure 1. Evaluation of histological sections revealed slow progression of hyperplastic lesions to malignant transformation and relatively low incidence of carcinomas between ages 6 and 30 weeks. Among the 71 mice analyzed, the only two cases of malignant carcinoma were detected in two 30-week-old mice. Therefore, for our next set of experiments aimed at assessing efficacy of Rapatar in preventing and/or delaying PCa development in *psPten–/–* mice, the age of 26 weeks was chosen as a starting point for Rapatar treatment.

### Rapatar impedes tumor development and progression

**Figure 1:**
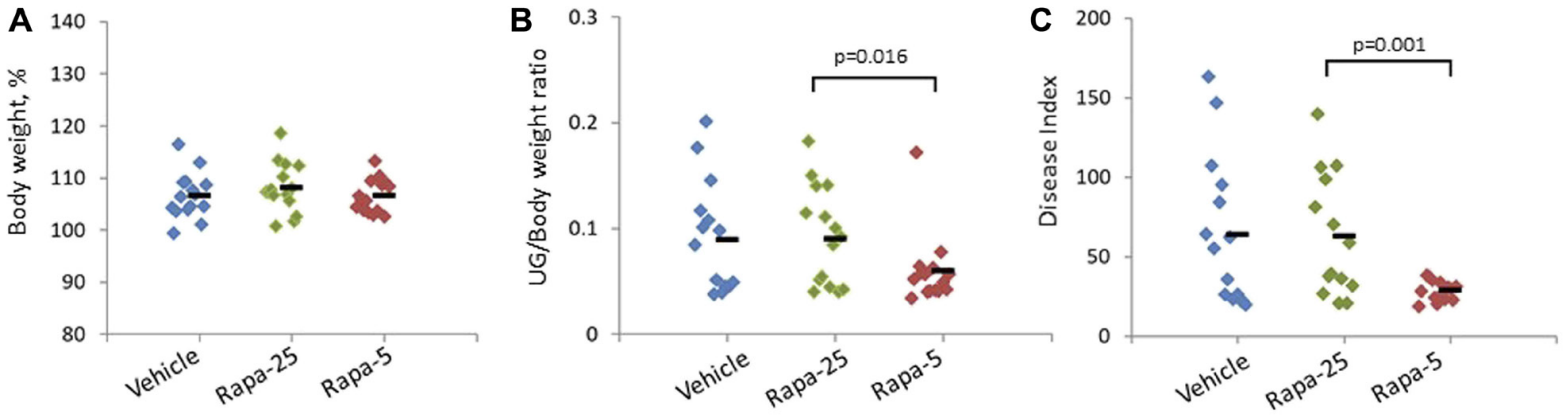
Effect of different Rapatar doses on body weight and PCa tumorigenesis in the *psPten* *–/–* mouse model. Groups were treated with vehicle or either 25 or 5 mg/kg Rapatar (Rapa-25 and Rapa-5 groups, respectively) for 8 weeks. Data for individual animals are shown, with the short horizontal black bar indicating the group mean value. (**A**) Administration of Rapatar does not affect animal growth. Body weights were measured at the end of the treatment period and plotted as a percentage of initial body weight. (**B**) Low dose Rapatar treatment reduces prostate tumor burden. The ratio of urogenital tract weight (UG) to body weight (BW) at the end of the experiment is plotted. (**C**) Low dose Rapatar treatment reduces overall urogenital disease. At the end of the experiment, Disease Index values were calculated as total histologic score^*^UG weight as described in {Kee, 2004 #123}.

To test the effect of different doses of Rapatar on PCa tumorigenesis, we treated groups of male *psPten–/–* mice with either 25 mg/kg (dose that slowed down tumor progression in *p53–/–* mice [32] or with a lower dose of 5 mg/kg of Rapatar beginning at 26 weeks of age (*n* = 15/group). Rapatar was administered via oral gavage 3 days/week for 8 consecutive weeks. Control mice were treated identically with vehicle. During the entire course of the experiment, mice were weekly weighed and palpated to record timing of tumor formation. As in our previous work using other mouse models, Rapatar administration did not appear to cause any general toxicity as illustrated by the similar changes in animal body weights in the control and Rapatar groups during the experiment (Figure 1A).

To assess how Rapatar affected the initiation and development of PCa, we first performed gross evaluation of the size of urogenital (UG) tract for each animal. At the completion of the 8-week treatment followed by 8-week post-treatment period, we dissected and measured the weight of the UG tract (including bladder, urethra, seminal vesicles, ampullary gland and prostate) and normalized it to body weight (BW) for each animal (Figure 1B). Interestingly, the UG/BW ratio was similar in mice that received higher dose of Rapatar (25 mg/kg) and in control vehicle-treated animals but was significantly lower in mice that received lower (5 mg/kg) doses of Rapatar. Overall, inhibition of prostate tumor growth was best when the lowest concentration of Rapatar was used, as it resulted in both significant reduction in UG/BW (*p* = 0.016, Student’s *t*-test) and more uniform UG/BW distribution (Figure 1B).

To further evaluate the degree of tumor development in control and Rapatar-treated groups, we performed histological examination of H&E-stained sections of anterior, dorsal, lateral and ventral lobes of prostates (AP, DP, LP and VP, respectively) collected at the end of the experiment. Since by this time none of mice developed advanced prostate carcinoma, in order to assess early stage histopathological differences between the groups, we applied scoring system that was specifically designed to discriminate between the subtle morphological changes at the initial stages of PCa development. For lobes of prostate we assigned a score of 0 to 3 for each of three parameters: (i) number of proliferating cells, (ii) presence of reactive stroma, and (iii) infiltration of immune cells. The AP, DP, LP and VP scores were then summed to generate a “total” score for each parameter. A detailed description of the criteria used for scoring is presented in Supplementary Table 1 and representative LP sections for each score are shown in Supplementary Figure 2.

As summarized in Table 1, scoring of prostate samples from the vehicle- and Rapatar-treated groups of mice described above showed that both doses of Rapatar significantly reduced the presence of proliferating cells detectable with H&E staining. The average “total” proliferation score was reduced from 8.27 ± 0.48 in the vehicle-treated group to 5.37 ± 0.36 and 5.86 ± 0.42 in the groups treated with 25 or 5 mg/kg Rapatar respectively (mean ± SEM; *p* < 0.001, one-way ANOVA). In contrast, there were no significant differences in the extent of immune cells. This suggests that Rapatar does not impact immune function at the doses used in this study. At the same time, scoring for the presence of reactive stroma revealed some unexpected differences. At higher dose of 25 mg/kg, Rapatar facilitated development of reactive stroma, as indicated by an increase in the total score for this parameter from 5.93 ± 0.53 in the vehicle-treated group to 7.53 ± 0.34 in the Rapa-25 group (*p* = 0.03, Student’s *t*-test). No increase in reactive stroma was seen in prostates of animals treated with low dose Rapatar.

**Table 1:** Average scores for histologic detection of the number of proliferating prostate epithelial cells, presence of reactive stroma and immune cell infiltration in AP, LP, DP and VP sections from psPten–/– mice treated for 8 weeks with vehicle or Rapatar (25 or 5 mg/kg; 15 mice/group)

Experimental Group	Proliferation					Presence of reactive stroma					Immune cell infiltration				
	AP	LP	DP	VP	Total	AP	LP	DP	VP	Total	AP	LP	DP	VP	Total
**Vehicle**	1.96	1.81	2.57	1.93	8.27	1.70	1.63	1.03	1.73	5.93	1.40	1.37	1.47	1.9	6.13
**Rapatar 25 mg/kg**	1.21	1.32	1.72	1.12	5.37^*^	2.07	2.10	1.37	2.0	7.53^^^	1.93	1.87	1.40	2.10	7.20
**Rapatar 5 mg/kg**	1.42	1.15	1.61	1.09	5.86^*^	1.55	2.03	1.07	1.13	5.98	1.77	1.63	1.43	1.50	6.33

“Total” is the sum of the AP, LP, DP and VP scores. ^*^significantly reduced proliferation in both Rapatar groups vs. vehicle group (*p* < 0.001, one-way ANOVA with Turkey post-hoc comparisons). ^^^significantly increased presence of reactive stroma in the 25 mg/kg Rapatar group vs. vehicle group (*p* = 0.03, Student’s *t*-test). *P*-values for all other comparisons between groups for a given parameter were > 0.05. Data are presents as mean values.

To quantify overall UG disease, a disease index (DI) value was calculated for each individual animal by multiplying the weight of the urogenital tract (UG weight, see above) by the sum of the total scores for all three histological parameters [39]. As shown in Figure 1C, mice treated with low dose Rapatar (5 mg/kg) showed a significant reduction in DI compared to higher dose Rapatar (*p* = 0.001, Student’s *t*-test). DI values in the Rapa-5 group were remarkably uniform, with no animals having a DI value over 50. In contrast, a DI value greater than 50 was observed in 8/15 and 7/15 mice in the vehicle and Rapa-25 groups, respectively. Overall, these data show that Rapatar impedes tumor development in tumor-prone ps*Pten–/–* mice, and although both tested does of Rapatar led to reduced proliferation in the prostrate, the greatest level of chemoprevention was achieved using low dose (5 mg/kg Rapatar, corresponding to 0.1 mg/kg rapamycin).

### Low dose Rapatar treatment reduces epithelial cell proliferation in the prostate without promoting stromal activation and autophagy

**Figure 2:**
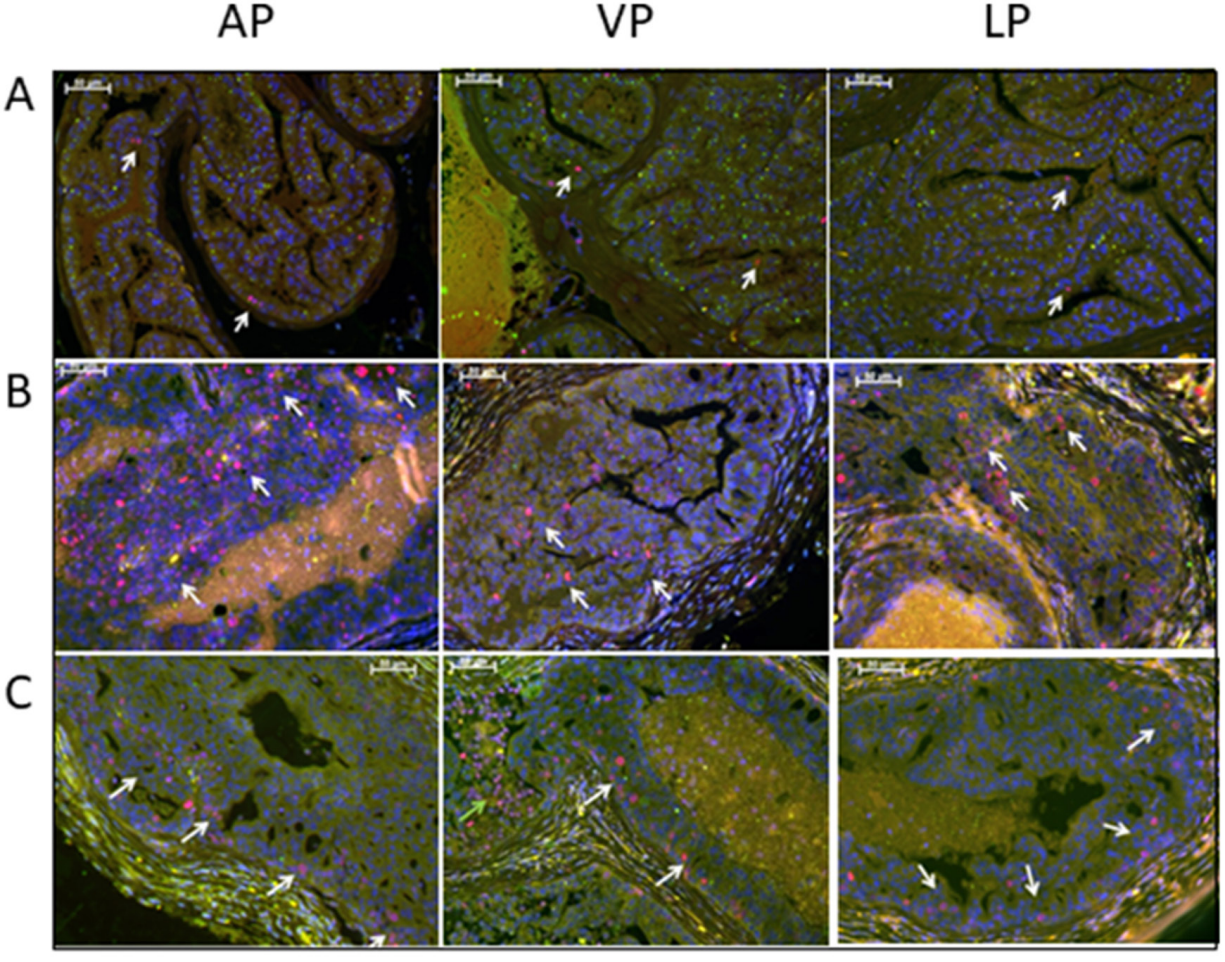
Treatment with low dose Rapatar reduces the number of proliferating cells in the prostate of PCa-prone *psPten–/–* mice. Representative anterior prostate lobe (AP), ventral prostate lobe (VP), and lateral prostate lobe (LP) sections stained with anti-Ki67 are shown for (**A**) *Pten-*positive mice, (**B**) *psPten–/–* mice treated with vehicle, and (**C**) *psPten–/–* mice treated with 5 mg/kg Rapatar. Red – Ki67, Blue-DAPI; White arrows indicate Ki67-positive nuclei.

To confirm our histological findings indicating that low dose of Rapatar reduced epithelial cell proliferation in the prostates of *psPten–/–* mice (Table 1), we immunoassayed prostate tissue sections with an antibody against the proliferation marker Ki67. Representative DP and LP sections from wild type (*Pten*-positive) and *psPten–/–* mice treated with either vehicle or 5 mg/kg Rapatar are shown in Figure 2. As expected, normal prostate tissue of wild type mice contained either none or very few Ki67-positive cells (Figure 2A), while PCa-prone *psPten–/–* mice had a dramatically increased number of such cells (Figure 2B). Samples from 5 mg/kg Rapatar-treated *psPten–/–* mice showed substantially lower levels of Ki67 staining compared to vehicle-treated *psPten–/–* controls (Figure 2C).

Despite having similar effects of epithelial cell proliferation in *Pten–/–* prostates, the tested doses of Rapatar showed differences in overall chemopreventive efficacy in this model. Increasing the dose of Rapatar from 5 to 25 mg/kg resulted in an increase in tumor burden (indicated by UG/BW ratio, Figure 1B) and overall DI (Figure 1C), suggesting that higher concentrations may cause side effects that counteract the drug’s anti-proliferative effects. Since reactive stroma plays a key role in prostate tumor development [40] and was found to be increased in *psPten–/–* mice treated with 25 mg/kg (but not 5 mg/kg) Rapatar (Table 1), we focused on potential effects of Rapatar on stromal cells as an explanation for the observed dose-dependence of PCa suppression.

**Figure 3:**
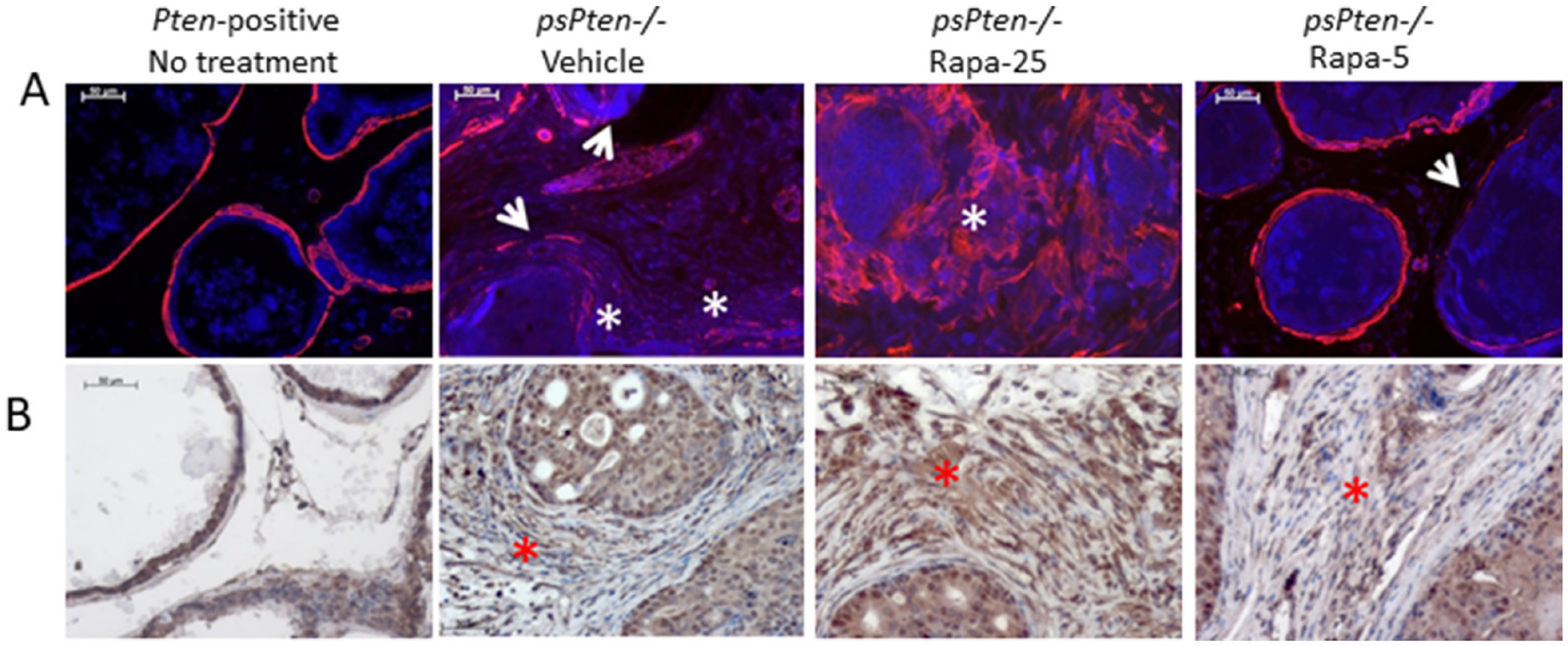
High dose (25 mg/kg) Rapatar treatment promotes development of reactive stroma and induces autophagy. Markers of reactive stroma (αSMA, panel **A**) and autophagy (LC3B, panel **B**) were evaluated by immunostaining of LP sections from untreated *Pten+/+* mice and *psPten–/–* mice treated with vehicle, 25 mg/kg Rapatar or 5 mg/kg Rapatar. In A, red staining indicates αSMA, blue is DAPI co-stain. White arrowheads indicate interruptions in αSMA staining and white asterisks show areas of strong staining for αSMA-positive activated fibroblasts. In B, brown staining indicates LC3B expression; red asterisks highlight areas of strong LC3B expression.

Changes in stromal characteristics during the process of tumorigenesis involve fibroblast-to-myofibroblast differentiation leading to accumulation of cancer-associated fibroblasts (CAFs) [41]. During this process, CAFs undergo metabolic reprogramming and facilitate tumor growth by secreting factors that promote proliferation of tumor cells and stimulate cell motility and metastasis [42]. The process of fibroblast-to-myofibroblast differentiation can be assessed by immunostaining prostate tissue with anti-smooth muscle α-actin (αSMA), the most common marker of activated fibroblasts [43]. As shown in Figure 3A, in normal prostate tissue from *Pten-*positive mice, αSMA is localized exclusively in the thin layer of smooth muscle cells surrounding each gland. PCa progression in *Pten–/–* mice leads to disintegration of the smooth muscle layer and development of αSMA-positive activated stroma (Figure 3A, Vehicle). Treatment of *Pten–/–* mice with high dose 25 mg/kg Rapatar appears to facilitate this process, as illustrated by representative LP sections showing significant disintegration of the smooth muscle layer and strong αSMA staining in the prostate stroma (Figure 3A, Rapa-25). In contrast, treatment of *Pten–/–* mice with low dose (5 mg/kg) Rapatar largely prevented these changes; the smooth muscle layer was well preserved and αSMA staining was similar to that seen in normal (*Pten-*positive) prostate samples (Figure 3A, Rapa-5).

There is substantial experimental evidence indicating that metabolic reprogramming of CAFs is accompanied by an increase in autophagy [44]. While under normal homeostatic conditions autophagy is an essential and beneficial catabolic mechanism, its overactivation in tumors promotes tumor cell survival (reviewed in [45]). To test whether the increased development of reactive stroma in the prostate caused by high dose Rapatar treatment positively correlates with the occurrence of autophagy, we immunostained the same prostate sections used for αSMA staining with antibodies against autophagy marker LC3B. As expected, the intensity of LC3B staining was positively correlated with the scale of reactive stroma development, being pronounced in prostates of *Pten–/–* mice treated with 25 mg/kg Rapatar but barely detectable in those treated with vehicle or low dose (5 mg/kg) Rapatar (Figure 3B).

Together, this data illustrates that rapamycin can have different effects on epithelial and stromal cells depending on the dose used. Specifically, while both the low and high doses of Rapatar used in our study (5 and 25 mg/kg, respectively) were equally effective in suppressing proliferation of prostate epithelial cells, the low dose did not induce development of reactive stroma and autophagy to the same extent as the high dose. We propose that these dose-dependent side effects underlie the differences in chemopreventive efficacy observed for different Rapatar doses.

### High dose Rapatar treatment promotes development of reactive stroma through feedback activation of Akt

Emerging studies indicate that the mTOR signaling pathway is regulated by complex processes, including several feedback loops which may be activated by mTORC1 inhibition. Thus, the downstream target of mTORC1, S6K, suppresses activity of the second mTOR complex, mTORC2, by phosphorylating one of its components, Rictor. mTORC2 plays a critical role in phosphorylation-mediated activation of AKT; consequently, inhibition of mTORC1 by rapamycin releases the negative feedback on PI3K/Akt signaling, resulting in activation of a pro-survival pathway [46] that has been linked to the fibrogenic process in several tissues [47–50]. This led us to hypothesize that the development of reactive stroma may be facilitated by feedback activation of Akt, and that the scale of Akt activation may be dependent on rapamycin dose.

**Figure 4:**
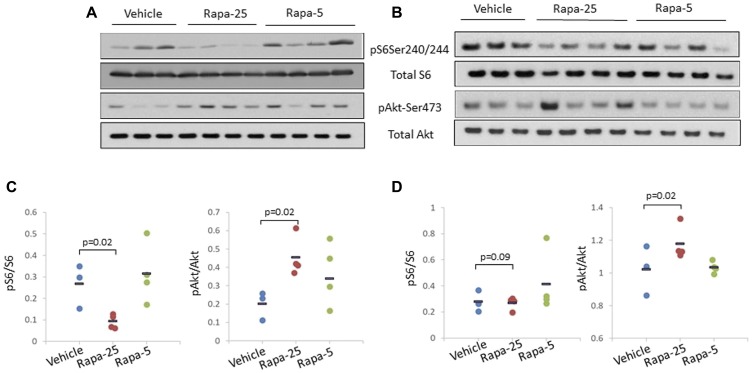
High dose (25 mg/kg) Rapatar treatment of *psPten–/–* mice leads to increased Akt activation in the liver and the prostate. Western blot analysis of whole cell extracts (25 ug protein per lane) prepared from the livers (**A**) and prostates (**B**) of individual mice treated for 8 weeks with vehicle (*n* = 3), 25 mg/kg Rapatar (*n* = 4) or 5 mg/kg Rapatar (*n* = 4). Membranes were probed with antibodies detecting activated (phosphorylated) and total S6 and Akt proteins. (**C**) and (**D**) Quantitation of ratio of activated to total protein for S6 and Akt in livers and prostates respectively based on Western blot signals. Data for individual animals are shown, with the short horizontal black bar indicating the group mean value.

To test this hypothesis, we analyzed various components of these regulatory pathways in liver and prostate samples from *psPten–/–* mice treated with two different doses of Rapatar (5 mg/kg or 25 mg/kg) for 8 weeks as described. At the end of the treatment period, liver and prostate tissues were collected for preparation of whole cell extracts for Western blot analysis (Figure 4A and 4B respectively). This showed that in both tissues analyzed, 25 mg/kg Rapatar inhibits S6 phosphorylation more effectively when compared to vehicle-treated animals (*p* = 0.02 and 0.09 in liver and prostate, respectively; Student’s *t*-test) than 5 mg/kg dose that showed no significant difference with vehicle-treated group (*p* = 0.5 and 0.8, respectively; Figure 4C and 4D, left panels). However, the low and high doses of Rapatar had different effects on Akt activity: increased phosphorylation of Akt was observed both in livers and prostates from the Rapa-25 group (*p* = 0.02 compared to vehicle-treated mice, Students *t*-test) but not in those from the Rapa-5 group mice (*p* = 0.4 and 0.5, respectively; Figure 4C and 4D, right panels). These data suggest that low doses of Rapatar may only minimally block S6K-dependent phosphorylation of S6, and thus, unlike high doses, may not be sufficient to relieve feedback activation of Akt, which is achieved by using higher doses.

**Figure 5:**
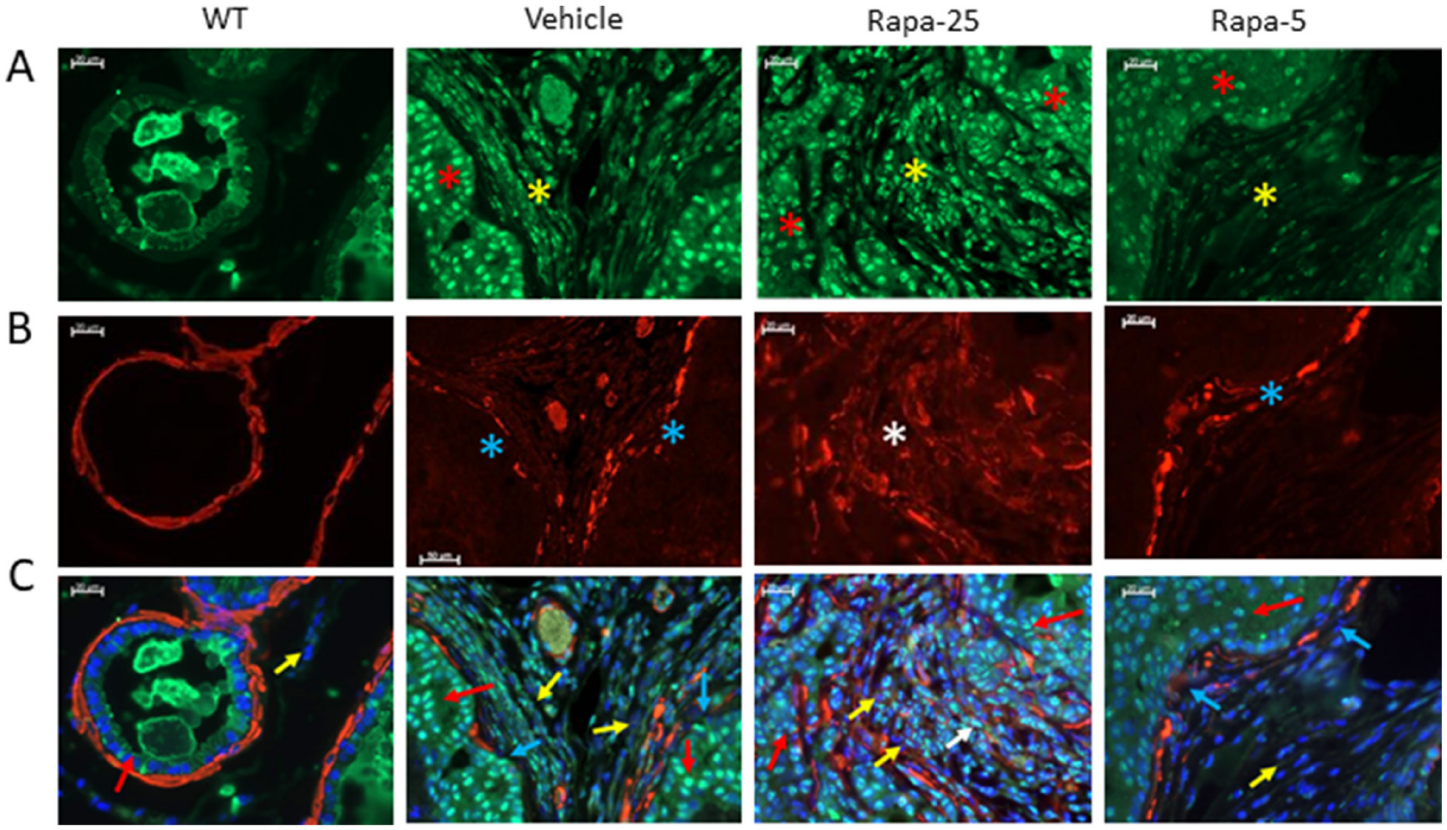
Dysregulation of TGFβ signaling correlates with development of reactive stroma in prostates of *psPten* *–/–* mice treated with high dose Rapatar. Immunostaining of pSMAD2 (panel **A**, green) and SMA (panel **B**, red) was performed on LP sections from wild type (WT) mice and *psPten–/–* mice treated for 8 weeks with vehicle, 25 mg/kg Rapatar or 5 mg/kg Rapatar. Panel (**C**) shows both markers overlaid with DAPI (blue) staining of nuclei. In A, red and yellow asterisks indicate nuclear stain of pSMAD2 in epithelium and stroma, respectively. In B, blue asterisks indicate areas of smooth muscle layer disintegration and white asterisks indicate appearance of smooth muscle actin in activated stroma. In C, Red arrows point out epithelial cells, yellow arrows point out stromal cells, blue arrows indicate the smooth muscle layer.

To fully test our hypothesis, we next sought to uncover the mechanism by which feedback activation of Akt promotes development of reactive stroma. Numerous previous studies suggest that the formation of reactive stroma in the prostate is associated with deregulated TGFβ signaling leading to fibroblast-to-myofibroblast transformation [40, 51]. TGFβ, which plays an important role in maintaining adult tissue homeostasis, participates in crosstalk with several different signaling pathways including the PI3K/AKT/mTOR pathway (reviewed in [52]. To test whether Rapatar-induced Akt activation correlates with activation of TGFβ, we co-immunostained prostate sections with anti-pSMAD2 (a marker of TGFβ activation) and αSMA. No nuclear staining of pSMAD2 was detected in the epithelium or stroma of normal prostate sections from wild type mice (Figure 5, WT). In control *psPten–/–* animals, nuclear signal from pSMAD2 was detected in both epithelial and stromal cells; however, this was not accompanied by significant alterations in the smooth muscle layer and few αSMA-positive fibroblasts were detected (Figure 5, Vehicle). In *psPten–/–* mice treated with high dose (25 mg/kg) Rapatar, we observed very strong nuclear staining of pSMAD2 in the glandular epithelium and stroma, which coincided with complete destruction of the peri-glandular smooth muscle layer and massive appearance of strongly SMA-positive fibroblasts (Figure 5B, white asterisk). These changes were all substantially reduced or absent in prostate samples from the low dose (5 mg/kg) Rapatar group: nuclear staining of pSMAD2 was reduced in the prostate epithelium and completely absent in stromal cells (Figure 5C, yellow arrows), there were only occasional alterations of smooth muscle layer integrity (Figure 5C, blue arrows), and there were very few, if any, SMA-positive fibroblasts. In summary, this analysis established a positive correlation between increased Rapatar dose, activation of TGFβ, activation of Akt, development of reactive stroma/induction of autophagy, and reduced PCa-preventive outcome. This suggests that the greater chemopreventive efficacy of low vs. high doses of Rapatar in our experiments was due to greater impacts of the high dose on mTOR regulatory pathways leading to development of reactive stroma, induction of autophagy, and other side effects that counteract the anti-proliferative effect of the drug. Overall, these findings provide a possible explanation for the past clinical failure of mTOR pathway inhibition as a chemopreventive strategy and suggest that use of lower inhibitor doses could lead to substantially better outcomes.

## DISCUSSION

Given the integral role that the mTOR pathway plays in regulating cell growth and proliferation, it is not surprising that its over-activation is characteristic of many types of cancers due to the selective advantage it provides to cancer cells. Inhibition of mTOR by rapamycin analogues (rapalogs) was evaluated as an anti-cancer strategy in clinical trials for renal cell carcinoma [53–55], hematopoietic malignancies [56, 57], ovarian cancer [58] and others (reviewed in [59]). However, while receiving FDA approval for renal cell carcinoma treatment, the efficacy of rapalogs in the clinic was significantly more modest than anticipated based on pre-clinical studies. This may be explained, at least in part, by complex and often contradictory results obtained in pre-clinical studies. For example, the effect of rapamycin can vary depending on mouse strain, sex, age, dose, route of administration, schedule of administration (continuous vs. intermittent), etc [21, 60–62]. This variability likely reflects the complexity of the mTOR signaling pathway and differential sensitivity of its components to rapamycin. First, of the two mTOR complexes (mTORC1 and mTORC2), only mTORC1 is effectively inhibited by rapamycin [48]. Second, the same dose of rapamycin suppresses phosphorylation of different mTORC1 substrates with different efficiencies; low doses that completely suppress S6K phosphorylation induce only partial inhibition of 4E-BP1 phosphorylation [63–65]. In addition, inhibition of mTORC1 relieves several negative feedback loops resulting in activation of PI3K, RAS/MAPK and/or mTORC2, which oppose the effects of rapalogs on protein biosynthesis and cell cycle progression [66–69]. The lack of success for rapalogs in clinical trials led to a shift in focus towards development of combination therapies using rapalogs together with various Akt, PI3K or autophagy inhibitors [70]. This approach has its own drawbacks, however, as many of these drugs demonstrate significant toxicity (reviewed in [71]).

More promising results were obtained using rapalogs for cancer prevention rather than treatment [72]. Rapamycin was shown to significantly delay the onset of tumor development in several models of cancer-prone mice [23, 32, 73]. Efficacy of rapamycin as a cancer preventive agent is also supported by clinical studies in which patients receiving rapamycin as an immunosuppressant after renal transplantation showed a decrease in cancer incidence [74, 75]. Nevertheless, use of rapamycin cancer prevention also has significant limitations since its continuous administration was associated with various adverse side effects in both pre-clinical and clinical studies [27–29].

In this work, we revisited the possibility of using a rapalog for cancer prevention based on the hypothesis that low doses of a highly bioavailable, nano-formulated form of rapamycin (i. e., Rapatar) might be sufficient to suppress tumorigenesis without inducing adverse side effects. Using the *psPten–/–* mouse model of PCa, we showed that continuous administration of Rapatar for 8 weeks delayed development and progression of PCa and that this delay was associated with suppression of prostate epithelial cell proliferation. While both tested doses of Rapatar (25 and 5 mg/kg) were equally effective in reducing proliferation in the prostate, evaluation of the overall extent of UG disease demonstrated that the low dose of Rapatar (5 mg/kg, Rapa-5) was most effective in suppressing tumor progression. We found that high dose of Rapatar promoted development of reactive stroma and autophagy that may counteract the beneficial anti-proliferative effect of the drug. This explains the difference in chemopreventive efficacy of high vs. low Rapatar doses in our study and is in line with several previous reports of dose-dependent rapamycin efficacy in different contexts [76]. Thus, while it could remain true that rapalogs may not be clinically viable options for cancer treatment (which in most cases will require high doses), our results suggest that Rapatar may still be considered as a cancer preventive drug if used at very low concentrations.

From a mechanistic standpoint, our data suggest that higher doses of rapamycin promote development of reactive stroma and autophagy, which at least partially, may result from rapamycin-induced mTORC2-dependent Akt activation leading to deregulation of TGFβ signaling. In agreement with this, it was reported that Rictor/mTORC2 signaling mediates TGFβ-induced fibroblast activation and kidney fibrosis [3]. Alternatively, Akt may induce reactive stroma via downregulation of TGFβ receptor, leading to increased production of TGFβ Franco, 2011 #339}.

Our data also show that the amount of reactive stroma present in the prostate correlates with stronger development of autophagy. While under normal conditions autophagy is important for maintaining cellular homeostasis and metabolism, its induction in stroma may result in secretion of factors that promote cancer cell survival, a phenomenon known as the reverse Warburg effect [77]. This may be directly promoted by high doses of rapamycin [78–80] or may develop in response to increased TGFβ production by reactive myofibroblasts [81]. Regardless of the mechanism of induction of autophagy (which we cannot discriminate at this point), our experiments consistently showed higher levels of autophagy in prostates of animals treated with higher dose of Rapatar.

**Figure 6:**
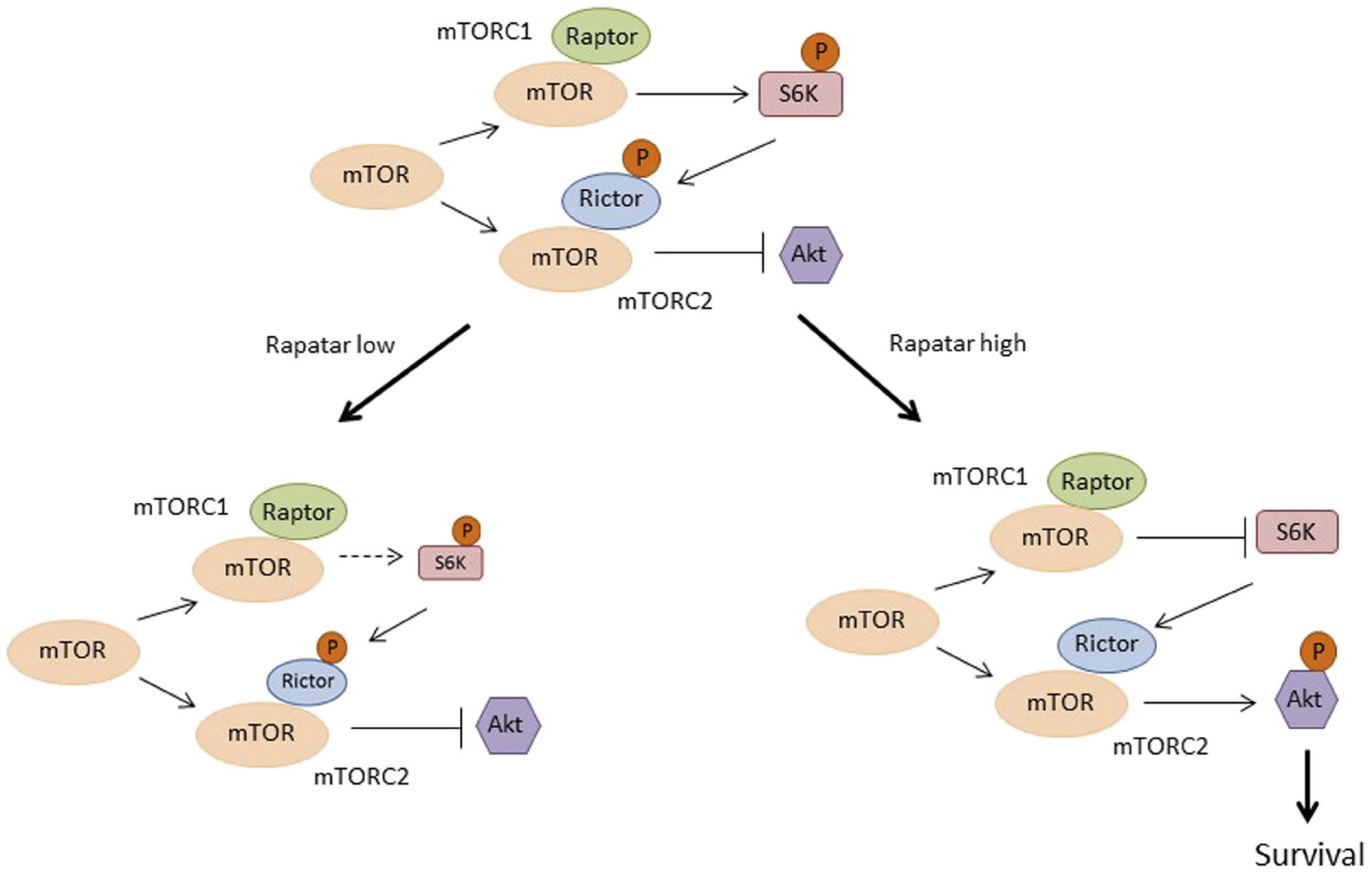
Proposed model explaining the differential tumor preventive effects of low and high doses of Rapatar on mTOR signaling in the *psPten–/–* mouse model. See details in the text.

Taken together with what is known regarding mTOR regulatory and signaling pathways, our findings allow us to propose a model for the differential effects of low and high doses of Rapatar, which is schematically presented in Figure 6. The model suggests that low dose Rapatar causes moderate suppression of S6K, which is sufficient for inhibition of proliferation but is not enough to release inhibitory effect of S6K on mTORC2; therefore, Akt is not activated (Figure 6, lower left panel). High dose Rapatar completely blocks S6K activity, thereby relaxing suppression of the negative feedback loop such that Akt becomes activated (Figure 6; lower right panel). Akt activation promotes formation of reactive stroma and autophagy, and thus contributes to tumor progression.

In conclusion, we have demonstrated that a novel formulation of rapamycin (Rapatar) can achieve chemopreventive efficacy at low doses that avoid undesirable side effects. This supports the possibility of reviving clinical development of mTOR inhibitor-based approaches for cancer prevention with particular focus on the importance of drug dose.

## MATERIALS AND METHODS

### Animals, breeding and genotyping

All animal experiments were conducted in accordance with the guidelines of the Institutional Animal Care and Use Committee (IACUC) of Roswell Park Comprehensive Cancer Center. Animals were housed in ventilated cages under standard conditions with food and water available *ad libitum*. B6.129S4-*Pten^tm1Hwu/J^* mice (transgenic mice containing loxP sites flanking exon 5 of the *Pten* gene, *Pten^flox/flox^*) and B6. Cg-Tg*(Pbsn-Cre)^4Prb/Nci^* mice (transgenic mice expressing Cre recombinase in prostate epithelium postnatally [34]) were obtained from the Experimental Tumor Model Shared Resource at Roswell Park. To generate mice with prostate epithelium-specific deletion of the *Pten* gene, female *Pten^flox/+^* mice were crossed with male *PB-Cre4*^+^ *Pten^flox/+^* mice. The male progeny were genotyped by PCR using DNA obtained from tail biopsies as described previously [82] to identify *Cre+; Pten^Flox/Flox^* experimental mice (referred to herein as *psPten–/–* mice).

### Rapatar administration

Male *psPten–/–* mice (15/group) received Rapatar at 25 or 5 mg/kg (corresponding to, 0.5 and 0.1 mg/kg rapamycin, respectively) starting at 26 weeks of age. Control mice received vehicle. All mice were negative for the presence of prostate tumors by palpation at the start of treatment. Rapatar was administered via oral gavage 3 days/week for 8 consecutive weeks. Animals were euthanized 8 weeks after completion of treatment (i. e., at 42 weeks of age) and prostates were dissected and used for histological analyses.

### Histopathological and immunohistochemical analysis of prostate tumors

Mouse UG tracts consisting of bladder, urethra, seminal vesicles, ampullary gland and prostate were excised and weighed. Individual lobes of the prostate were dissected, including observable tumors. Tissues were fixed in 10% neutral buffered formalin for 24 hours and then processed in an automated processor (Leica ASP 300) and embedded in paraffin using a LEICA EG 1150H embedding unit according to the manufacturer’s protocols. Five micron-thick sections were obtained using a rotary microtome (LEICA RM 2235) and stained with hematoxylin and eosin (H&E) according to a standard protocol (DAKO Coverstainer). Histopathological examination was performed using a Zeiss AxioImager A1 microscope equipped with an Axiocam MRc digital camera. Morphological changes in the anterior, dorsal, lateral and ventral lobes of prostates were blindly evaluated by a mouse pathologist and graded for each of 3 parameters (proliferation rate, presence of reactive stroma and infiltration of immune cells) according to the grading system described in Supplementary Table 1. Scores for each lobe were summed to generate a total score for each individual mouse for each parameter. A Disease Index value was calculated for each mouse by multiplying total score by the UG weight.

Proliferating cells were visualized in prostate tissue sections using indirect immunofluorescence with a rabbit monoclonal anti-Ki67 antibody (Clone SP6, Thermo Scientific, 1:200 dilution) and Cy3-conjugated anti-rabbit antibody (Jackson ImmunoResearch, 2 μg/ml). Nuclear DNA was counterstained with DAPI. Green channel image was captured in order to exclude auto-fluorescence.

Smooth muscle actin (SMA) and LC3B were visualized in prostate sections by immunofluorescence and immunohistochemistry, respectively, after heat-induced antigen retrieval in citrate buffer (pH 6.0). For SMA staining, Cy3-conjugated anti-SMA mouse monoclonal antibody (Sigma-Aldrich, 1:1000 dilution) was used. After incubation with the antibody, slides were washed and mounted with ProLong Diamond anti-fade reagent with DAPI (ThermoFisher). For LC3B staining, rabbit polyclonal anti-LC3B antibody (Novus Biologicals, 1:200 dilution) and HRP-conjugated donkey anti-rabbit antibody (ThermoFisher, 1:1000 dilution) were used. Slides were counterstained with hematoxylin.

For pSMAD2 staining, anti-Phospho-Smad2 (Ser465/467) (138D4) rabbit monoclonal antibody and AlexaFluor488-conjugated donkey anti-rabbit antibody (Jackson ImmunoResearch, 1:500 dilution) were used.

### SDS-PAGE and Western blotting

Prostate tissue was homogenized using a Polytron PT 10-35 GT Kinematica homogenizer in RIPA buffer containing protease and phosphatase inhibitors (Sigma-Aldrich). Homogenates were incubated at 4°C for 20 minutes and then cleared by centrifugation (16,000 × g for 20 minutes at 4°C). Total protein concentrations in whole cell extracts were determined using the Pierce BCA Protein Assay Kit. Whole cell extracts (25 μg protein per lane) were resolved using NuPage 4-12Bis-Tris Protein Gels (Invitrogen), transferred to PVDF membrane, and probed with antibodies against Akt, pAkt (Ser473), ribosomal protein S6 and pS6 (Ser240/244) (Cell Signaling). Peroxidase-conjugated goat anti-rabbit or goat anti-mouse (Jackson Immuno Research Laboratories) were used as secondary antibody. Proteins were visualized by Denvilles’s HyGlo chemiluminescent detection reagent and quantitated using ImageJ software.

### Statistical analyses

Student’s *t*-test and one-way ANOVA were used for two groups and multiple groups’ comparisons respectively. *P*-values < 0.05 were considered statistically significant.

## SUPPLEMENTARY MATERIALS



## Abbreviations

mTOR: Mechanistic Target of Rapamycin
PTEN: Phosphatase and Tensin Homolog
PCa: prostate cancer
4E-BP1: Eukaryotic translation initiation factor 4E-binding protein 1
BPH: benign prostate hyperplasia
TRAMP: Transgenic Adenocarcinoma Mouse Prostate
UG: urogenital
BW: body weight
AP: anterior prostate
DP: dorsal prostate
LP: lateral prostate
VP: ventral prostate
DI: disease index
αSMA: smooth muscle α-actin
