# Supplementary material for: Superior cancer preventive efficacy of low versus high dose of mTOR inhibitor in a mouse model of prostate cancer
Source: Oncotarget. 2020 Apr 14;11(15):1373–87. doi: 10.18632/oncotarget.27550 (PMC7170500; doi:10.18632/oncotarget.27550)
Supplement: Supplementary file 1 [file oncotarget-11-1373-s001.pdf]

Superior cancer preventive efficacy of low versus high dose of mTOR inhibitor in a mouse model of prostate cancer

SUPPLEMENTARY MATERIALS

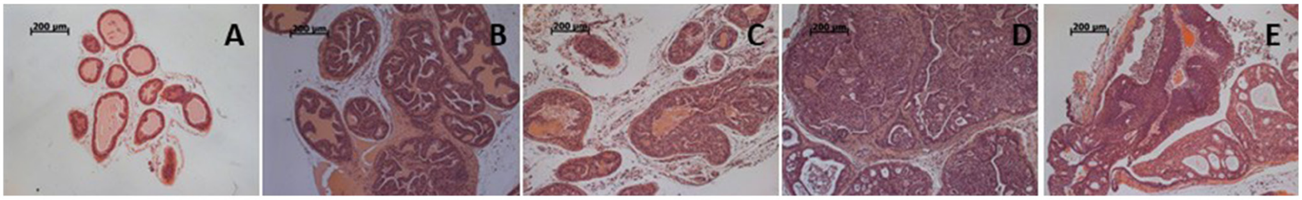

Supplementary Figure 1: Representative transverse sections of prostate tissue illustrating our histopathology-based system for scoring prostate cancer development and severity. Prostate sections were prepared from untreated *psPten–/–* mice of various ages. (**A**) Prostatic glands lined with normal looking epithelium: cells of uniform size with basophilic nuclei and eosinophilic cytoplasm; only a few low and simple protrusions of the epithelium are seen in the lumen filled with secretion (pink); score 1. (**B**) More numerous and more complex single layer hyperplastic protrusions into the lumen of the glands resulting in partial cribriform and stellate obstruction of the lumen; score 2. (**C**) Uniformly thicker epithelial lining of a gland (right) or thicker lining on one side and simple single layer lining on the other side of the same acinus (left); presence of some interstitial infiltration; score 3. (**D**) Prostatic gland carcinoma with extensive growth of epithelial cells and loss of glandular arrangement of the cells; appearance of cells of various sizes, hyperchromic nuclei, cell crowding and piling; score 4. (**E**) Prostatic carcinoma in which the malignantly transformed neoplastic cells (upper left) differ significantly in appearance and growth pattern from the cells of benign hyperplasia (lower right) with no intermediate morphology between the two areas; score 5.

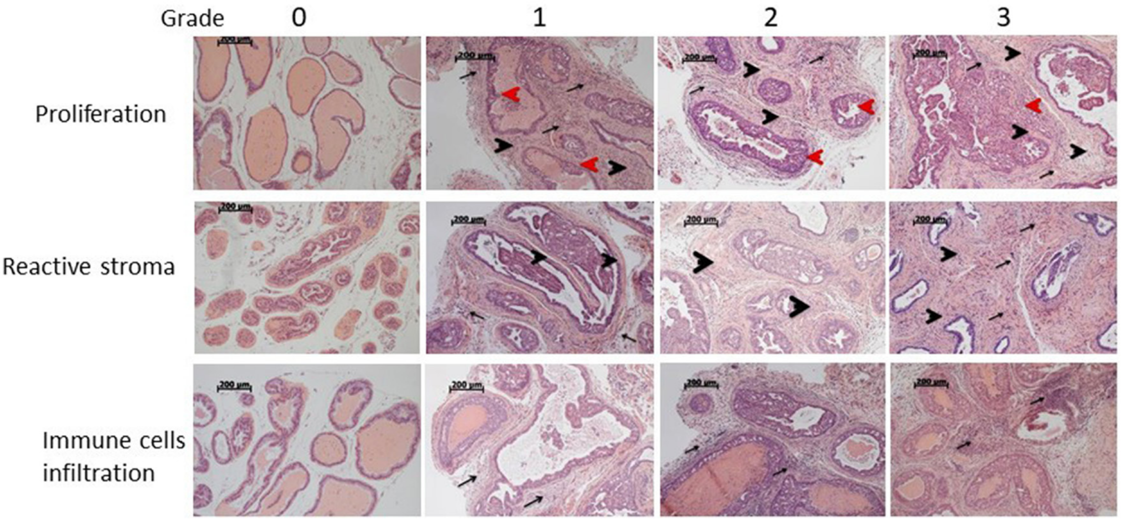

Supplementary Figure 2: Representative sections of prostate lateral lobes showing various degrees (Grade 0–3, left to right) of the number of proliferating cells (top row), presence of reactive stroma (middle row) and infiltration of immune cells (bottom row). Red arrowheads indicate the hyperplasia of the lining epithelial cells; black arrowheads mark the reactive stromal reaction; thin black arrows indicate infiltrating immune cells.

Supplementary Table 1: Criteria for histological grading of proliferation, presence of reactive stroma and immune cell infiltration in H&E-stained prostate tissue sections

	Proliferation	Presence of reactive stroma	Immune cell infiltration
Grade 0: "normal"	Acini with variably sized lumens are lined by simple cuboidal epithelium with centrally located nuclei. Pale intraluminal secretion as well as sparse stroma (delicate basement membrane of acini and thin fibrils).	Acini surrounded by delicate basal membrane and scant reticular stroma visible as single thin strands in seemingly empty spaces between the acini.	Single transitory infiltrative mononuclear or lymphoid cells present.
Grade 1	Hyperplasia of epithelial cells (morphologically indistinguishable normal cells) at single sites or along the whole circumference of the acini resulting in epithelium several cells thick (red arrow). The amount of connective tissue (black arrow) and of infiltrating cells (thin black arrows) is considerably more pronounced.	Minimal stromal reaction; the fibrillar and cellular connective tissue elements surrounding the acini and basal membrane of most acini are approximately twice as thick as the simple epithelial lining (black arrows).	5 to 10 infiltrative mononuclear or lymphoid cells present.
Grade 2	Interepithelial hyperplasia of epithelial cells resulting in thicker walls of acini along most of their circumference, minimal protrusions into the lumen and rounded or stellate transverse sections (red arrow); inside these protrusions and accumulations of cells, infoldings of the basal membrane or blood vessel branches were not observed.	Moderate stromal reaction; the connective tissue elements form thicker cuffs around the acini, contain more cells and show coarser and thicker fibrillar bundles.	20 to 30 infiltrative mononuclear or lymphoid cells present (thin black arrow); cells are dispersed.
Grade 3	Papillary infoldings and prominent intraluminal ridges of hyperplastic epithelium (red arrow) with connective tissue cores and sinusoids in them; partial obstruction of the lumen of acini, with the epithelial lining being taller on one side than the other, but this growth remains confined within the surrounding thickened stromal layer (black arrow).	Considerable stromal reaction with prevailing interstitial stromal component over the adjacent acini.	Numerous (> 30) infiltrative mononuclear or lymphoid cells present; cells are often more densely packed.

*see Supplementary Figure 2.
